# Sex differentiation in Atlantic cod (*Gadus morhua* L*.*): morphological and gene expression studies

**DOI:** 10.1186/1477-7827-10-47

**Published:** 2012-06-18

**Authors:** Trine Haugen, Fernanda FL Almeida, Eva Andersson, Jan Bogerd, Rune Male, Katrine S Skaar, Rüdiger W Schulz, Elin Sørhus, Tim Wijgerde, Geir L Taranger

**Affiliations:** 1 Institute of Marine Research, Nordnes, P.O. Box 1870, N-5817, Bergen, Norway; 2 Utrecht University, Science Faculty, Department of Biology, Padualaan 8, NL-3584 CH, Utrecht, The Netherlands; 3 University of Bergen, Department of Molecular Biology, HiB, Thormøllensgt. 55, N-5020, Bergen, Norway; 4 Embrapa Western Amazon, Rodovia AM-10, Km 29, PO Box 319, Manaus, AM-Brazil-69010-970, Brazil

**Keywords:** Atlantic cod, Sex differentiation, Aromatase, *cyp19a1a*, Anti-müllerian hormone, *amh*, *dax1*, *shp*, Masculinization

## Abstract

**Background:**

In differentiated gonochoristic species, a bipotential gonad develops into an ovary or testis during sex differentiation. Knowledge about this process is necessary to improve methods for masculinizing genetically female Atlantic cod for the subsequent purpose of producing all-female populations.

**Methods:**

Gonads were examined histologically in juveniles from 14 to 39 mm total body length (TL). Number and size of germ cells were determined in a subset of the samples. Relevant genes were cloned, and mRNA levels determined by qPCR of *amh*, *cyp19a1a*; *dax1 (nr0b2); shp (nr0b2a) and sox9b* in a mixed-sex and an all-female population ranging from 12–49 mm TL.

**Results:**

Individuals between 14–20 mm TL could be separated in two subgroups based on gonad size and germ cell number. Ovarian cavity formation was observed in some individuals from 18–20 mm TL. The mixed sex population displayed bimodal expression patterns as regards *cyp19a1a* (starting at 12 mm TL) and *amh* (starting at 20 mm TL) mRNA levels. After approximately 30 mm TL, *cyp19a1a* and a*mh* displayed a gradual increase in both sexes. No apparent, sex-dependent expression patterns were found for *dax1*, *shp* or *sox9b* transcripts. However, *shp* levels were high until the larvae reached around 35 mm TL and then dropped to low levels, while *dax1* remained low until 35 mm TL, and then increased sharply.

**Conclusions:**

The morphological sex differentiation in females commenced between 14–20 mm TL, and ovarian cavities were evident by 18–20 mm TL. Testis development occurred later, and was morphologically evident after 30 mm TL. This pattern was corroborated with sexually dimorphic expression patterns of *cyp19a1a* from 12–13 mm TL, and a male-specific increase in *amh* from 20 mm TL.

## Background

All female populations of Atlantic cod (*Gadus morhua*) are desired in aquaculture to prevent possible genetic impacts on wilds stocks from fertilized eggs spawned in cages. All female populations is even more beneficial if combined with triploidy, as triploid fish normally do not develop large gonads and secondary sex characters that are regarded negative in the grow-out phase in aquaculture [1,2]. All female populations are most commonly produced by using sperm from masculinized XX females [3,4]. However, in order to successfully masculinize fish, the timing of sex differentiation (time window when the fish are labile to phenotypical sex change), must be known.

Sex differentiation is the complex process of developing a functional testis or an ovary from a bipotential vertebrate gonad [5]. In fish, maternally transferred mRNAs, necessary for the formation and migration of primordial germ cells (PGCs), is segregated asymmetrically into the future PGCs [6]. The PGCs migrate towards the gonadal ridge [7], where they become enclosed by somatic gonadal cells [8]. The PGCs remain quiescent in the undifferentiated gonad for a period of time [9], before they start to proliferate and differentiate into oogonia or spermatogonia upon extrinsic cues. The timing of sex differentiation varies between teleost species, and it can occur very early as in medaka (*Oryzias latipes*) with increasing numbers of germ cells in presumptive females already at hatching [10], intermediate as in zebrafish (*Danio rerio*) (21–25 day post hatching) [11], or late in juvenile stages as in sea bass (*Dicentrarchus labrax*) (120–150 days post fertilization) [12,13] and Atlantic halibut (*Hippoglossus hippoglossus*) at approximately 38 mm fork length [14].

Some common features are observed during sex differentiation in different fish species. One is the up-regulation of expression of the *cyp19a1a* gene. Cyp19a1a protein catalyzes the conversion of androgen into estrogen, which drives ovarian differentiation [15-17]. Sex specific expression of *cyp19a1a* in the gonads has been observed for example in southern flounder (*Paralichthys lethostigmata*) [18] and Nile tilapia (*Oreochromis niloticus*) before and during sex differentiation [19,20].

Anti-Müllerian hormone (Amh) is well studied in mammals, but less so in teleosts. In mammals, Amh induces regression of the Müllerian duct during male sex differentiation, and also modulates the differentiation of Leydig cells by down-regulating the expression of several genes coding for steroidogenic enzymes [21], and it inhibits expression of the aromatase gene during sex differentiation [22]. Teleosts have no Müllerian duct, but Amh has been shown to inhibit steroidogenesis in adult zebrafish testis [23]. The undifferentiated gonad express *amh* at 17 and 21 dpf, with a male-biased expression starting at about 30 dpf in zebrafish [24,25], suggesting that Amh is important in male zebrafish sex differentiation. Male-biased overexpression has also been shown in other species, like Japanese flounder (*Paralichthys olivaceus*) [26] and rainbow trout (*Oncorhynchus mykiss*) [27,28].

The *dax1* gene (dosage sensitive sex-reversal adrenal hypoplasia critical region, chromosome x, gene 1) codes for a nuclear receptor protein, that amongst others represses the transcription of steroidogenic factor 1 (Sf1) in mammals [29], which in turn regulates the expression of many steroidogenic enzymes and genes involved in reproduction [30]. Dax1 has an essential role in fetal testis development in mice [31], and *Cyp19* aromatase expression is up-regulated when *Dax1* is disrupted [32]. In contrast, over-expression of this gene caused male to female sex reversal in humans [33]. In fish the role of Dax1 is less clear.

Shp (short heterodimer partner) is a nuclear receptor protein that belongs to the same subfamily of nuclear receptors as Dax1. It functions as a transcriptional co-repressor that inhibits the expression of steroidogenic genes by inhibiting the expression of *sf1,* thereby acting as a gonadal gatekeeper of male sexual maturation in mice [34]. The Shp protein might have similar functions in fish, as *shp* is highly expressed during early life stages in Nile tilapia (5 dph) and seemed to repress the activity of *sf1*[35]. Moreover, in rainbow trout, *shp* was highly expressed during early stages of sex differentiation, and then decreased twofold in both sexes [27].

Sox9 is a transcription factor containing the DNA-binding motif HMG, and is considered one of the more important genes related to sex differentiation in vertebrates [36]. In mammals, Sox9 has multiple functions such as cartilage formation and testis differentiation [37,38]. In zebrafish, two *sox9* genes have been identified: *sox9a* and *sox9b*[39]. Sox9a may have a role in testis, and Sox9b may have a role in ovary development, based on expression patterns in adults. Hence, in zebrafish, *sox9a* may be male biased and *sox9b* may be female biased. However, both sox9a and sox9b are expressed in chondrogenic cells of both sexes [39]. A sexually dimorphic *sox9* expression pattern was also indicated in medaka and rainbow trout [40,41].

Based on morphological studies, sex differentiation in Atlantic cod was initially reported to take place from approximately 27 mm total body length (TL) [42], and steroid treatment to induce masculinization was recommended from approximately 25 mm TL. But a more recent study demonstrated that exposure of Atlantic cod to 17-α-methyltestosterone in the diet from 12 mm to 25 mm TL onwards resulted in high proportion of hermaphrodites, suggesting that sex differentiation commenced earlier than at 27 mm TL [2]. We therefore wanted to carry out a combined morphological and molecular study on sex differentiation in Atlantic cod to study this process further.

We examined the morphological development of the early gonads in individuals from 14 to 34 mm TL. Moreover, in individuals from 12 to 49 mm TL we studied the expression of genes known to be involved in sex differentiation and early gonadal development in other teleosts species. Partial sequences of Atlantic cod *amh*, *dax1, shp* and *sox9* were obtained, specific real-time qPCR assays were developed and validated, and transcript levels were analyzed in the trunk (head and tail removed) of individuals from a normal, mixed sex population and from an all-female population produced with sperm from sex-reversed genetic females.

## Methods

The following experiment was approved by the National Animal Research Authority in Norway in advance of the experiment.

### Fish material

Origin: Three XX hermaphrodites (genotypic females) from a previous masculinization trial [2] and one XY male (genotypic male) were slaughtered, testis tissue was excised and carefully minced in separate sterile dishes, before dilution with Hanks Balanced Saline Solution (HBSS – modified; Sigma-Aldrich, St Louis, Missouri, USA) (3 ml milt/30 ml HBSS) and incubated on ice for 30 min. Approximately 250 ml of stripped eggs from two XX females were mixed and separated into 4 equal batches; three batches were fertilized with sperm from the three XX hermaphrodites, and one batch were fertilized with sperm from one normal XY male. The batches were incubated for 35 min before transfer into four separate 70 L incubators at ambient temperature (6.2 °C ± 0.1) and salinity (35‰). At four days post hatching (dph) (118.6 degree days) the larvae were transferred group-wise to start-feeding tanks (50 L) with a water temperature of 8.3 °C, which was raised to 12 °C over the following 10 days (14 dph). The tanks were supplied with algae paste and rotifers, and 33 dph the larvae received artemia, and were gradually weaned onto dry feed from 36 dph. The weaning diet was a commercial agglomerated diet (Ewos Aglo norse, Bergen, Norway) with a particle size range of 300–500 μm (for fish between 9–14 mm total body length; TL), and of 400–600 μm (for fish between 13–22 mm TL). All tanks were supplied with automatic feeders to ensure continuous feeding.

### Sampling

For gene expression analysis, approximately 50 individuals were sampled from both the mixed sex population and one of the all-female populations at each of the following time points: 66, 74, 79, 87 and 96 days post fertilization (n = 500 individuals). The average TL (± SD) at each sampling was 11.2 (±2.4); 18.4 (±4.9); 23.9 (±3.6); 30.4 (±3.8) and 43.7 (±4.0) mm respectively.

The fish were sedated in MS-222 (tricaine methanesulfonate, Finquel, Washinton, USA) (0.013 g/500 ml chilled seawater (SW)). TL was measured, and head and tail removed before the trunk was wrapped in pre-labeled aluminum foil and snap frozen in liquid nitrogen. The tissue was kept at −80 °C until analysis.

### RNA extraction and gene cloning

Total RNA was isolated from mature Atlantic cod testis using Trizol reagent (Invitrogen, Carlsbad, California, USA) according to established procedures. The RNA amount was quantified (NanoDrop Technologies, Wilmington, DE, USA) and its quality checked by agarose gel electrophoresis. cDNA was subsequently generated using a SMART RACE cDNA kit (Clontech, Mountain View, California, USA), according to the manufacturer’s instructions. Cloning of gene specific cDNA fragments was done by PCR using primers deduced from orthologous gene sequences or from sequence searches in an in-house expressed sequence tag (EST) database. All PCR primers are given in Table 1. The obtained fragments were subcloned in pCR4-TOPO (Invitrogen, Carlsbad, California, USA) and sequenced.

**Table 1:** **Primers for cloning of*amh, dax1, shp, sox9b*and*vasa*in Atlantic cod**

**Primer name**	**Direction**	Primer sequence*
amh_fw1	forward	5′-CGACCAGCAGGAGAGCTCCAGTACA -3′
amh_rv2	reverse	5′-CATGTTTTCCTTGACGTGGCTGAGG-3′
dax1_fw1	forward	5′-TGCAAAGCSGCSTCSSMRGTYCTGGYGAARAC-3′
dax1_rv2	reverse	5′-CCASCACGAGCARRGGYGCCCA-3′
dax1_fw3	forward	5′-GATACGCTTCGTGAAAAACGTGCCGTGTTTTCG-3′
dax1_rv 4	reverse	5′-GACGACCAGCTGGTGCTCGTGCGGAGC-3′
shp _fw1	forward	5′-ACTTTATGAAGAACTTGCCGGCGTTTAACCAGCTG-3′
shp_rv 2	reverse	5′-CAGCGATCAGTTTTCGCTGCTCCAGAAGTGCTG-3′
sox9_fw1	forward	5′-GGCTACGACTGGACNYTNGTNCCNATG-3′
sox9_rv2	reverse	5′-GGCAGGTACTGRTCRAACTCRTYGAC-3′
vasa_fw1	forward	5′-GGTGTCAACTTTGAYAARTAKGA-3′
vasa_rv2	reverse	5′-CCGGTTCTACCAATKCGRTGNACRTA-3′

*R = A or G, Y = T or C, S = G or C, M = A or C.

An Atlantic cod *amh* sequence was obtained using primers amh_fw1 and amh_rv2 to generate a 387 base pairs (bp) long PCR fragment that was verified as *amh* by sequence similarity (Genbank accession no HQ630631). Primers for *sox9* (sox9_fw1 and sox9_rv2) were predicted from an alignment of several fish *sox9* sequences and used for PCR amplification of a 653 bp long fragment that was verified by sequencing (Genbank accession no HQ630630). The sequence of *vasa* from tilapia, zebrafish and trout were aligned and used to predict primers vasa_fw1 and vasa_rv2. The primers produced a 1087 bp long fragment that was verified as *vasa* (Genbank accession no HQ630632). To clone a partial cod *dax1* cDNA sequence, degenerate primers dax1_fw1 and dax1_rv2 were designed, and PCR products of ~160 bp were sub-cloned and sequenced. Two different sequences were obtained: one sequence was highly similar to *dax1*, whereas the other sequence was highly similar to *shp* sequences. To obtain additional cod *dax1* cDNA sequences, specific primers dax1_fw3 and dax1_rv4 were designed and used for 3′-RACE in combination with the UPM and NUP primers, respectively (supplied in the SMART RACE kit; Clontech, California, USA), using 3′RACE-ready mature cod testis cDNA as template. PCR products of ~900 bp were generated, gel purified with QIAEX, TOPO cloned and sequenced (Genbank accession no HQ677835). In a similar way, specific primers shp_fw1 and shp_rv2 were designed to obtain additional cod *shp* cDNA sequences with 3′-RACE. Approximately 675 bp PCR products were generated, gel purified with QIAEX, TOPO cloned and sequenced (Genbank accession no HQ677836).

The cloning and development of real-time PCR assay of Atlantic cod *cyp19a1a* has been reported earlier (as *cyp19a1*) [43].

### Real time quantitative PCR (qPCR)

In order to obtain gene expression profiles from individual larvae, total RNA were isolated from frozen larvae trunks (−80 °C) using an Invitrogen iPrep^TM^ Purification Instrument (Invitrogen,Carlsbad, California, USA) and the IPrep ^TM^ Trizol ® PLUS RNA Kit (Invitrogen, Carlsbad, California, USA) according to the manufacturer’s instructions. Homogenization of the tissue was performed using 2 ml tubes containing zirconium oxide beads in a Precellys® 24 Homogenizer (Bertin, Villeurbanne, France).

RNA quantity and quality were determined by UV absorbance at 230, 260 and 280 nm using a NanoDrop® NP-1000 spectrophotometer (NanoDrop technologies, Wilmington, DE, USA). Samples with a 260/280 nm absorbance ratio outside the range of 1.7 – 2.1 were excluded from further analysis. On a subset of the samples (approximately 10%), RNA integrity was verified with a Bioanalyzer 2100 expert system (Agilent Technologies Inc., Santa Clara, USA) and all samples had RIN values from 8–10. Reverse transcription into cDNA took place using a reverse transcription core kit (RT-RTCK-05, Eurogentec, Searing, Belgium) according to the manufacturer’s instructions with 500 ng total RNA in 30 μl reaction volume. The cDNA was diluted 10-fold with nuclease free water.

Specific primers and probes for real-time, quantitative PCR analysis of Atlantic cod *amh*, *cyp19a1a*, *dax1*, *shp*, and *sox9b* mRNAs as well as for the reference gene *ef1α* are given in Table 1. They were all designed with Primer express software (Applied Biosystems, Carlsbad, California, USA), according to the manufacturer’s guidelines.

TaqMan PCR assays were performed in duplicate, using 96-well optical plates on an ABI Prism Fast 7900HT Sequence Detection System (Applied Biosystems, Carlsbad,CA, USA) using default settings (95 °C for 20s, followed by a 40 cyckles of 95 °C for 1 s and 60 °C for 20s). For each 25 μl PCR reaction 2.5 μl cDNA was mixed with 200 nM fluorogenic probe, 900 nM sense primer, 900 nM antisense primer in 1xTaqMan Fast Universal PCR Master Mix (Applied Biosystems, Carlsbad, California, USA). Gene expression data were calculated relative to the smallest and youngest fish, using the ΔΔCt method as described in detail previously [44].

### Gonad histology

For the evaluation of gonad histology, Atlantic cod ranging from 14 to 34 mm TL were obtained from a commercial hatchery (Sagafjords, Bergen, Norway). These fish were reared under the same temperature and feeding regime and exhibiting growth rates comparable to the fish used for the gene expression experiment. The fish were sedated as described above, the length was measured, and the fish were euthanized with MS-222 (tricaine methanesulfonate, Finquel, Washinton, USA) (0.04 g/500 ml SW). The juveniles were individually fixed either in 5% v/v PBS-buffered glutaraldehyde, or in 4% w/v (RNase-free) PBS-buffered paraformaldehyde at 4^0^ C overnight. After dehydration, the glutaraldehyde-fixed samples were embedded in Technovit 7100 resin (Heraus Kultzer Wehrheim, Germany) while the paraformaldehyde-fixed samples were embedded in RNase-free paraffin (Histowax 56.58 °C, VWR International, Norway), according to conventional techniques. Serial longitudinal sections of 2 (plastic embedded) or 4 (paraffin embedded) μm thickness were collected and mounted consecutively on glass slides. Some individuals were transversally embedded and sectioned; for this purpose they were first decalcified - by immersion in 80% formic acid at 40^0^ C during 48 h – for facilitating sectioning. The serial histological sections were stained with 1% w/v toluidine blue 2% w/v borax staining solution. Each gonad was analyzed according to morphological features, such as shape, presence, size and number of germ cells and presence of an ovarian cavity.

In eight individuals (14 to 20 mm TL), the total number of germ cells was quantified in plastic embedded serial sections. The length of the gonad was measured by summing up the total number of sections (2 μm) in which gonad tissue was observed. For estimating germ cell number per gonad, the diameter of germ cell nuclei was determined in 8 juveniles using an ocular with a scale bar. Based on the germ cells’ average diameter (7.1 ± 0.04 μm; n ~ 20/juvenile), germ cells were counted in every third section of 2 μm, to avoid double counting.

In order to visualize germ cells, *vasa* mRNA was detected by *in situ* hybridization. A cod *vasa* PCR product (405 bp) was generated using primers vasa_ISH1 and vasa_ISH2 (Table 2) and cod testis cDNA as template. The PCR product was gel purified and 300 ng served as template for digoxigenin (DIG)-labeled cRNA probe synthesis by *in vitro* transcription (Roche Molecular Biochemicals) according to the manufacturer’s instructions.

**Table 2:** Primers and Probes for qPCR and ISH

**Transcript**	**Primer or probe**	**Sequence (5′–3′)**
*amh*	forward primer	GTCAGGCCAGCGAGAGCA
	reverse primer	AGGGCGACAACACATACGTTTC
	probe	[FAM]-CATCTGCAGGTGCAGGAACACTATATGC-[TAMRA]
*cyp19a1a*	forward primer	ACAACAACAAGTACGGCAGCAT
	reverse primer	GTAGAGGAGCTGCTGAGGATGAG
	probe	[FAM]-CGGCGTATGGATCAA-[MGB]
*ef1á*	forward primer	GCCCCTCCAGGACGTCTAC
	reverse primer	ACGGCCCACGGGTACTGT
	probe	[FAM]-AGATCGGCGGTATTG-[MGB]
*dax1*	forward primer	TGGTGGCGCAGCTCTTCT
	reverse primer	GCAGCACCTCTTCCATGTTGA
	probe	[FAM]-CAAGCCCGTGATCGGCGCC-[TAMRA]
*shp*	forward primer	GCGGGCGTCGGTAAACTTA
	reverse primer	CGCGTACTCCTTCGGACTCA
	probe	[FAM]- CAAACCGCCAAACCTCTTGAGACAGGGCCAAAC -[TAMRA]
*sox9b*	forward primer	AAGAAGCCGAGCGCTTAAGG
	reverse primer	CCGCCTCGGTTGGTATTTG
	probe	[FAM]-TTCAACACAAAAAGGACCACCCGGACT-[TAMRA]
*vasa*	forward primer	T3Rpps- TGACCTGGACCAGCTTCTGCTCCACGTCA
	reverse primer	T7Rpps- CAAGTTTGCTCATGGGACCTGCGTGCGT

For *vasa in situ* hybridization, RNase-free paraformaldehyde fixed, paraffin (Histowax 56.58 °C, VWR Interantional, Norway) embedded individuals were used*,* as described in [45], using sense and antisense cRNA probes at a concentration of 200 ng/ml of hybridization buffer. Ribonuclease A (2 μg/ml; Sigma-Aldrich, St. Louis, USA) treatment was performed in RNase buffer (0.01 M Tris, 0.5 M NaCl, 0.005 M edetic acid [EDTA], pH 7.5). As positive control, the same procedure for *vasa* mRNA *in situ* hybridization was performed on sections of mature cod testis.

### Statistics

The germ cell number (per gonad) and gonad length (μm) were tested for statistical differences using a non-parametric Mann–Whitney *U* test (*p* < 0.05) (Statistica 9, StatSoft inc. Tulsa, USA).

## Results

### Gonadal histology and germ cell morphometry

**Figure 1:**
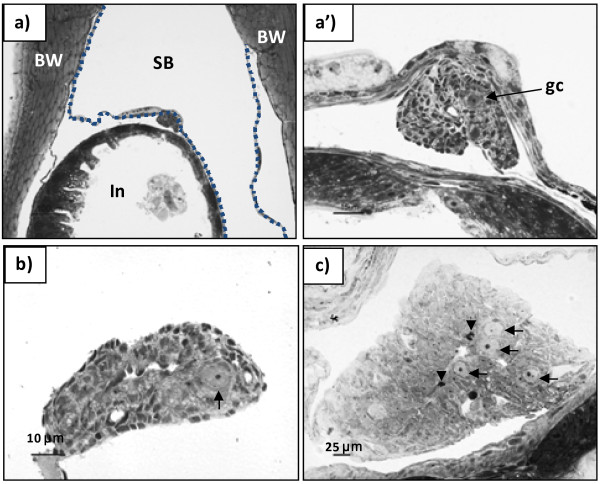
**Differentiating gonad of Atlantic cod, presumptive female.** Transverse plastic section (2 μm) stained with Toluidine blue/Borax solution. **a**) and **a’**) formation of an ovarian cavity in a 16 mm TL juvenile. a): scale bar represents 100 μm; a’): scale bar represents 15 μm); **b**) gonad with an ovarian cavity in a 18 mm TL juvenile; **c)** ovary in a 30 mm TL juvenile. SB – swim bladder; In – intestine; BW – body wall; gc – germ cell. The stippled line identifies the ventral part of the swimming bladder in a). Arrows indicate primordial germ cells; arrowheads in c) indicate mitotic figures.

**Figure 2:**
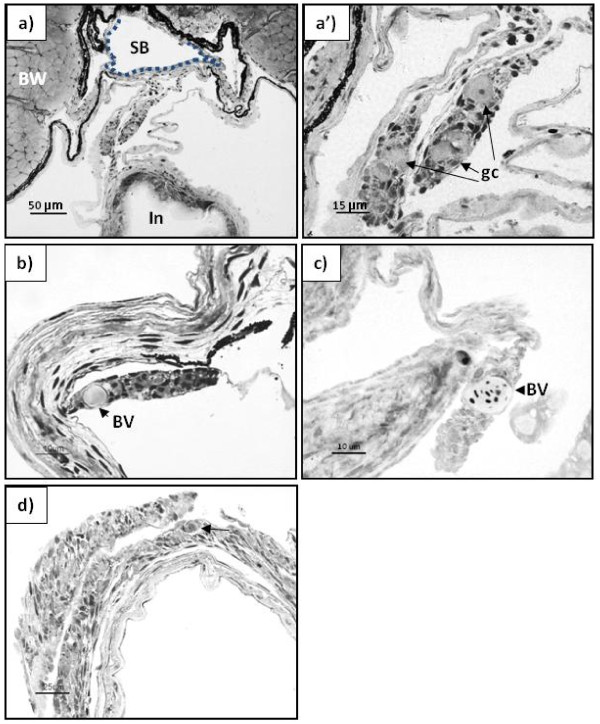
**Differentiating gonad of Atlantic cod, presumptive male.** Transverse plastic section (2 μm) stained with Toluidine blue/Borax solution. **a**) and **a’**) Undifferentiated gonad in a 14 mm TL juvenile; **b**) Early testis in a 18 mm juvenile (scale bar represents 10 μ); **c**) Early testis in a 34 mm juvenile (scale bar represents 10 μm); **d**) Testis of a 32 mm juvenile (scale bar represents 25 μm). BV – blood vessel; arrows indicate primordial germ cells.

**Figure 3:**
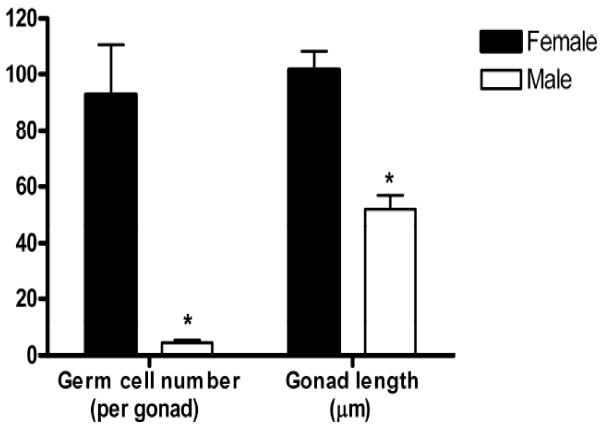
**Number of germ cells per gonad.** Total number of germ cells per gonad and gonad length of Atlantic cod juveniles (n = 8) ranging between 14 to 20 mm TL. Asterisks denote significant difference, *P* < 0.05 (Man-Whitney non-parametric test).

In juvenile Atlantic cod, gonad tissue is located in the dorso-caudal region of the abdominal cavity, ventral of the swim bladder (Figures 1a and 2a), and connected to the dorsal body wall by a thin connective tissue, the mesogonadium. In individuals between 14 to 18 mm TL, the gonads consisted of connective tissue, small blood vessels and large germ cells (7.1 ± 0.04 μm diameter), with no apparent difference between individuals as regards somatic elements and germ cell morphology (Figures 1a’ and 2a’). A more detailed analysis on a subsample of eight individuals between 14–20 mm TL, revealed that the total number of germ cells per gonad and the gonad size was either high or low (Figure 3). Moreover, the group with the high number of germ cells showed several mitotic figures in the germ cells and a large gonad size, while the group with few germ cells also showed few mitotic figures in the germ cells and small gonads.

Histological examination of gonadal development in individuals in the size range 18–20 mm TL revealed the first signs of ovarian cavity formation in five of the examined individuals: gonadal somatic tissue started to grow out laterally (Figure 1a and 1a’), arched back to the gonad, and eventually fused in a zipper-like pattern. The fusion progressed rostro-caudally, creating the ovarian cavity in the stroma of the forming ovaries (Figure 1a and 1b). In similarly sized fish without signs of ovarian cavity formation, a large blood vessel was observed in the proximal region of the gonad (Figure 2b and 2c); lateral outgrowths were absent. From 20 mm TL onwards, the gonads grew considerably, resulting in an enlargement of the ovarian cavity concomitant with an increase in the number of germ cells in the ovaries, while an elongation of the gonad was seen in presumptive males (Figures 1c and 2d respectively).

### *In situ* hybridization

**Figure 4:**
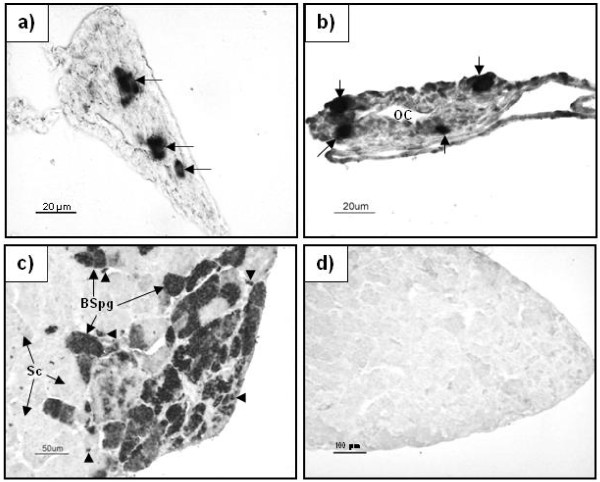
***In situ*hybridization for*vasa*mRNA in Atlantic cod gonads.a**) Testis tissue of a fish of 34 mm TL; **b**) ovarian tissue of a fish of 32 mm TL, OC – ovarian cavity; **c**) In a mature (adult) testis (positive control); **d**) In a maturing testis using sense probe (negative control). Arrows indicate positive oogonia/spermatogonia in a) and b); arrowheads show type A spermatogonia in c, BSpg – type B spermatogonia; Sc – spermatocytes.

To identify and verify the presence of germ cells in the developing gonads with an independent approach, we performed *in situ* hybridization for *vasa* mRNA. The signal for *vasa* was very strong in the germ cells in all gonads of individuals up to 34 mm TL (Figure 4a and 4b). Maturing testis was used as a positive control (Figure 4c). Expression of *vasa* was strong in spermatogonia, weak in spermatocytes, and absent in spermatids and spermatozoa. No signal was observed in sections incubated with the sense probe (Figure 4d).

### Gene expression during sex differentiation

**Figure 5:**
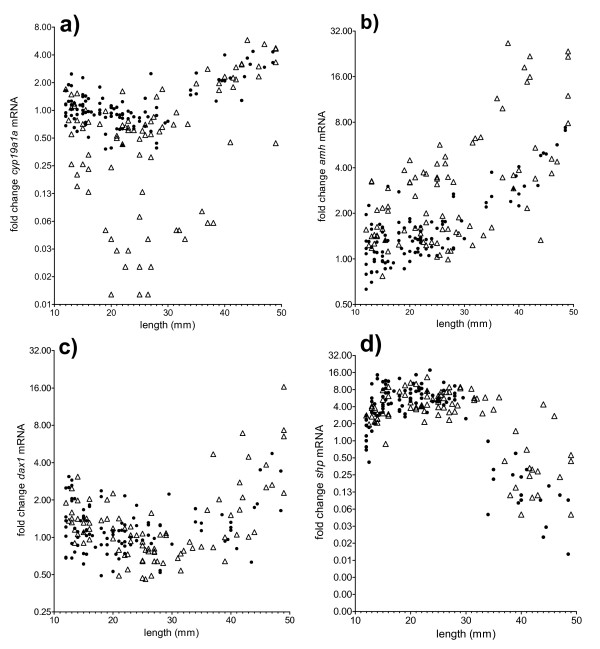
**Gene expression profiles in early life stages of Atlantic cod.** Gene expression profiles in early life stages of mixed sex (open triangles) and all-female (closed circles) Atlantic cod; **a**) *cyp19a1a*; **b**) *amh*; **c**) *dax1*; **d**) *shp*. Data are presented on a logarithmic scale as fold change compared to the smallest and youngest fish.

Throughout most of the sampling period (12–40 mm TL), the expression of *cyp19a1a* mRNA was higher in the all-female population and in approximately half of the individuals of the mixed sex population, compared to the other half of the individuals of the mixed sex population (Figure 5a). However, an increase was observed in *cyp19a1a* mRNA expression from around 30–35 mm TL, which started from different levels in the two subgroups of the mixed sex group.

The transcript levels of *amh* showed no bimodality until around 20 mm TL, and the mixed sex group and the all female group had overlapping values in that size range, while an apparent bimodal pattern was evident from 20 mm TL onwards in the mixed sex group. From around 30–35 mm TL increasing levels were seen in both modals of the mixed sex group, and in the all female group (Figure 5b).

*dax1* mRNA levels showed a size dependent pattern (Figure 5c). All individuals exhibited low levels with some variation until approximately 30–35 mm TL, after which a up-regulation of *dax1* mRNA levels was recorded in all individuals. The mRNA levels of *shp* also changed with the size but displayed a pattern opposite to that of *dax1*: up to 30–35 mm TL, levels were high but then declined (Figure 5d).

The expression of *sox9b* showed a gradual twofold increase with size (see Additional file 1: Figure S1), Expression levels of the reference gene *ef1α* were relatively stable throughout the sampling period for all individuals (see Additional file 2: Figure S2).

## Discussion

In Atlantic cod, the undifferentiated gonad is positioned ventral of the swim bladder in the caudal part of the body cavity, has very few germ cells and was observed in juveniles ranging from 14 to 18 mm TL. The first morphological sign of ovarian differentiation was observed in individuals of 18 to 20 mm TL, when an empty space formed in the stroma of the ovaries, known as the ovarian cavity. In the majority of gonochoristic teleosts examined, the appearance of an ovarian cavity is the earliest morphological sign of ovary formation [9].

In the size range of 14–20 mm TL, two subgroups were found that differed in germ cell number and gonad size, indicating ongoing morphological sex differentiation during this period. A common early distinction between ovary and testis is based on the number of germ cells present in the early gonad, since oogonia generally show earlier elevated mitotic activity and enter earlier into meiosis than spermatogonia [46]. The somatic part of the gonads appeared to be histologically undifferentiated until 18 mm TL in all individuals examined. Taken together, our morphological observations support the notion that individuals with a high number of germ cells before ovarian cavity formation were females whereas those with a low germ cell number most likely were males. These findings are in line with the observation of high mitotic activity of germ cells in developing ovaries of many teleost species [9]. In a more recent work on three-spined stickleback (*Gasterosteus aculeatus*), an increase in the number of germ cells in gonads of female but not of male fry preceded the appearance of traditional morphological criteria for sex differentiation [47].

In the current study, based on these considerations, individuals in the size range of 18–34 mm TL with no apparent ovarian cavity and a low number of germ cells were considered to be males. These presumptive males had small filament-shaped gonads, with few undifferentiated germ cells, but with large blood vessels in the proximal region of the gonad. The formation of blood vessels at the proximal or distal regions of the gonad is considered an indicator of the early testis in several fish species [46,48].

Despite differences in germ cell numbers between presumptive males and females in the size range of 14–20 mm TL, further germ cell differentiation was not observed, indicating that the somatic differentiation of ovaries in individuals of approximately 18–20 mm TL preceded germ cell differentiation, except for their increased proliferation in females. This is in accordance with the conclusions of Nakamura et al. [46], where morphological differences in the stroma may give a more correct indication of the timing of the sex differentiation than germ cell morphology.

To confirm the identity of the germ cells, we performed vasa *in situ* hybridization as *vasa* mRNA is restricted to germ cells [49-51]. In individuals of 32–34 mm TL, we observed a very strong *vasa* signal in the germ cells.

 Our results confirmed that Atlantic cod is a differentiated gonochoristic species, where the sexually undifferentiated gonad develops directly into a testis or an ovary [42]. Our study provides evidence that morphological gonad differentiation in Atlantic cod occurs earlier in females than in males, as generally found in other primary gonochoristic teleosts [5,9]. Different from the results presented here, an earlier report described ovarian cavity formation in Atlantic cod at 27 mm TL [42]. This divergence might be related to significant growth differences between the two studies.

Our use of an all-female population compared with a normal mixed sex population allowed studying potential sexually dimorphic gene expression patterns during the period of sex differentiation. During the period from 12–20 mm TL, *cyp19a1a* appeared to have a sexually dimorphic expression pattern in the mixed-sex population in parallel with the observed morphological female sex differentiation. The all-female population displayed a relatively high and less variable *cyp19a1a* expression in this size range, corresponding to the levels of the highest modal of the mixed sex group.

A bimodal expression pattern of *cyp19a1a* was apparent already from 12–14 mm TL in the mixed-sex group, suggesting that molecular sex differentiation already commenced in this size range before morphological indications of female sex differentiation. In a similar manner, a sexually dimorphic pattern in the expression of *cyp19a1b* in the brain of rainbow trout populations before and during the early morphological gonad differentiation has been reported [27,52].

From around 30–35 mm TL onwards, an increasing *cyp19a1a* expression was observed both in the presumptive females and presumptive males, as well as in the all-female population. This may indicate that Cyp19a1a also has a role in male development in this size range, and may be related to up-regulation of steroidogenesis in both sexes.

In the size interval from 12–20 mm TL, no sexually dimorphic expression pattern was evident for *amh*. However, from around 20 mm TL until the end of the experiment, *amh* displayed an apparent sexually dimorphic expression pattern, as indicated by low transcript levels in the all-female population and the bimodal pattern in the mixed sex group. This pattern suggests a role for Amh in testis differentiation starting from 20 mm TL onwards.

The further increase of *amh* mRNA observed after 30 mm TL together with increasing *cyp19a1a* mRNA levels in the presumptive males may be related to a possible function of aromatase and estrogens in stimulating stem cell proliferation, while increased *amh* mRNA levels may at the same time have prevented the onset of spermatogenesis as shown in Japanese eel (*Anguilla japonica*) [53,54]. Furthermore, in the all-female population and the presumptive females of the mixed sex population, there was a gradual increase of *amh* mRNA levels from around 35 mm TL onwards, although at lower levels than in the presumptive males, suggesting a role for *amh* also in females. This is corroborated by the finding that *amh* is expressed in granulosa cells in zebrafish [24].

*dax1* and *shp* did not show an apparent sexually dimorphic pattern in our study. However, a size dependent pattern was evident, with a low expression of *dax1* from the first samples at 12 mm TL, which persisted until approximately 35 mm TL, when *dax1* expression increased in most individuals. The opposite was true for *shp*, which was expressed initially at high levels, with a drop to low expression level after approximately 35 mm TL. One possible reason for the lack of an apparent sexually dimorphic expression of *dax1* may be that this gene is also expressed in many other tissues apart from the gonads [35,55], which may mask potential sex-dimorphic expression in the gonads. In Nile tilapia, *dax1* and *shp* showed no sexually dimorphic expression during sex differentiation, and the expression of *dax1* was weak in the early stages (5–10dph)) and then significantly up-regulated between 10–15 dph [35].

The high initial levels of *shp* found here, followed by a drop to low expression levels after approximately 35 mm TL, are consistent with findings in rainbow trout [27], where *shp* was found to belong to a group of genes showing high expression during early ovarian and testicular development, but decreasing in both sexes when gonads had differentiated and gametogenesis was about to commence. This decrease in *shp* transcript levels during/after sex differentiation may reflect that Shp-mediated repression of other hormone receptors might fade with completion of sex differentiation. Moreover, Shp suppressed the expression of steroidogenic enzymes in mouse Leydig cells [34], thus increasing *cyp19a1a* and decreasing shp are consistent with increased steroidogenic activity, also in the presumptive males after around 35 mm TL.

A phylogenetic analysis of teleost *sox9* sequences deposited in the NCBI databank together with Atlantic cod sequences ( Additional file 3: Figure S3) obtained from the recent genome sequence [56], revealed two subtypes that both are clearly distinct from *sox8*, similar to the situation in other teleosts such as zebrafish [39], medaka [42] and rainbow trout [43]. The Atlantic cod *sox9* transcript studied in this communication is a *sox9b* variant, although the classification is somewhat unclear due to variable naming among the databank entries. The two *sox9* variants are probably co-orthologues that are partially subfunctionalized [42], but possibly with species-specific expression pattern in gonads as indicated in medaka and zebrafish [39,42].

The *sox9* variant that was analysed in the current study was thus classified as *sox9b*, and was found to have no sexually dimorphic pattern between 12–49 mm TL. However, there was a gradual increase in the expression of this gene throughout the sampling period. This was also the case in tilapia during sex differentiation [19], but in this species, *sox9* was up-regulated in male gonads after sex differentiation. In our experiment, a possible explanation for the lack of male specific expression of *sox9b* could be that the use of the whole trunk may have masked any sexually dimorphic expression pattern in the gonads, since Sox9 is also an important gene for cartilage development [39]. At this stage, there will be a substantial cartilage development due to high growth rates in general.

## Conclusions

**Figure 6:**
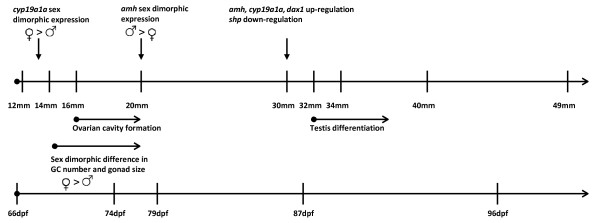
**The timing of the sex differentiation process in Atlantic cod.** Schematic presentation of the timing of the sex differentiation process in Atlantic cod based on histological events and mRNA expression profiles of *cyp19a1a* and *amh*. dpf is days post fertilization; GC is germ cell.

Our results suggest that sex differentiation in Atlantic cod has already commenced at around 12 mm TL, resulting in a sex-dimorphic expression pattern of *cyp19a1a*. From 14 mm TL, there was a difference in the proliferation of germ cells and in gonad size in presumptive females. The expression profiles of *amh* suggest that male differentiation commenced at approximately 20 mm TL, but apart from the large blood vessel at the proximal region of the gonad, testicular differentiation was not morphologically evident until later. At around 34–35 mm TL, there was a change in the expression profiles of *cyp19a1a*, *amh*, *dax1* and *shp*. The increase of *cyp19a1a*, *amh*, *dax1* and the decrease of *shp* are compatible with up-regulation of genes encoding nuclear receptors and steroidogenic genes in males, possibly in context with estrogen-mediated expansion of the spermatogonial stem cell population. The main findings are schematically summarized in Figure 6.

Overall, the data suggest that treatments to masculinize Atlantic cod should commence before 18 mm TL, when the first morphological signs of female development were noted, and possibly as early as 12 mm TL, or earlier, as the first molecular signs of sex dimorphic development were noted from the start of the experiment. A body size of 12 mm TL correspond to the time when Atlantic cod normally are weaned from live prey diet to formulated feed, and thus a time when androgens or other compounds can easily by administered in a controlled manner. Application of e.g. androgens or aromatase inhibitor treatments before this body size would normally imply treatment in water which can be more difficult to control in terms of dosage and uptake. The suggested time window for androgen treatment as suggested in the current paper is supported by a recent study in Atlantic cod [2]. The study revealed that androgen treatments starting at 12 mm TL and lasting until 25 mm TL were very efficient in inducing sex reversal resulting in high proportions of hermaphrodites, while the same treatments in the size range of 12 to 20 mm TL were less effective, and treatments in the size range of 12 to 16 mm TL was not effective at all [2]. This suggests that the androgen treatment must cover the entire period when female differentiation was noted in the current study to be efficient in inducing sex reversal.

## Competing interests

The authors declare they have no competing interests.

## Authors’ contributions

TH: planned and carried out the study, sampling, gene expression data treatment. Drafted the MS. FFLA: histological analysis and In Situ Hybridization. Helped draft MS. EA: carried out and analyzed the gene expression studies. Helped draft MS. JB and KSS: carried out the cloning and developed quantification of gene expression systems. RM: cloning and phylogenetic analysis. RWS: analyzed the histology and ISH, fund raising. Helped draft MS. ES: carried out and developed quantification the gene expression studies. TW: carried out parts of the histology. GLT planned the study, fund-raising, project leader. Helped draft MS. All authors have read and approved the final version.

## Supplementary Material

Additional file 1 **Figure S1.** Gene expression profiles of *sox9b* in early life stages of mixed sex (open triangles) and all-female (closed circles) Atlantic cod. Data are presented on a logarithmic scale as fold change in mRNA compared to the smallest and youngest fish. Click here for file

Additional file 2 **Figure S2.** Scatter plot of C_t_ values for *ef1α* mRNA from the mixed sex (open triangles) and all-female (closed circles) Atlantic cod individuals. Data are from quantitative real time PCR. All samples had similar template RNA concentrations loaded into the qPCR reaction. Click here for file

Additional file 3 **Figure S3.** Phylogenetic analysis of Sox9 amino acid sequences from different teleost species depicted as trees generated by the Neighbor-Joining (NJ) method (left) and Maximum Likelihood (ML) method (right). The bar represents 5% divergence between sequences. The sequences group into two large classes as noted as a and b to the right. The sequences are named according to appearance in the NCBI databank. Only apparently full length sequences were included in the alignment. *Clarias gariepinus* Sox9a ADJ96868, *Clarias gariepinus* Sox9b ADJ96869, *Cynoglossus semilaevis* Sox9a ACY05958, *Cyprinus carpio* Sox9b AAX56088, *Danio rerio* Sox9a AF277096, *Danio rerio* Sox9b AF277096, *Danio rerio* Sox9b AF277097, *Danio rerio* Sox8 AAX73357, *Dicentrarchus labrax* SOX9 CBN81190, *Epinephelus akaara* Sox9 AAT77677, *Epinephelus coioides* Sox9 ACT10337, *Gasterosteus aculeatus* Sox9a AAQ62978, *Gasterosteus aculeatus* Sox9b AAQ62979, *Monopterus albus* Sox9a1 AF378150, *Monopterus albus* Sox9a2 AF378151, *Odontesthes bonariensis* SOX9 AAP84605, *Oncorhynchus mykiss* SOX9 BAA24365, *Oncorhynchus mykiss* SOX9a2 AAG43497, *Oreochromis aureus* SOX9 ABY66377, *Oryzias latipes* SOX9b AAX62151, *Oryzias latipes* SOX9a AAX62152, *Paralichthys olivaceus* Sox9 ACO40490, *Poecilia reticulata* Sox9 ABG77973, *Salmo salar* Sox9 ACN10975, *Takifugu rubripes* Sox9 AAL32172. The Atlantic cod sequences were obtained through Blast search at The Cod Genome Project web site [57] and translated from the Gadus morhua Sox9 ENSGAUG00000009261 (apparent complete open reading length that include the primer sites used in this communication) and Gadus morhua Sox9v ENSGAUG00000015623. Click here for file
