# Supplementary figures and images for: Sex differentiation in Atlantic cod (Gadus morhua L.): morphological and gene expression studies
Source: Reprod Biol Endocrinol. 2012 Jun 18;10:47. doi: 10.1186/1477-7827-10-47 (PMC3433390; doi:10.1186/1477-7827-10-47)

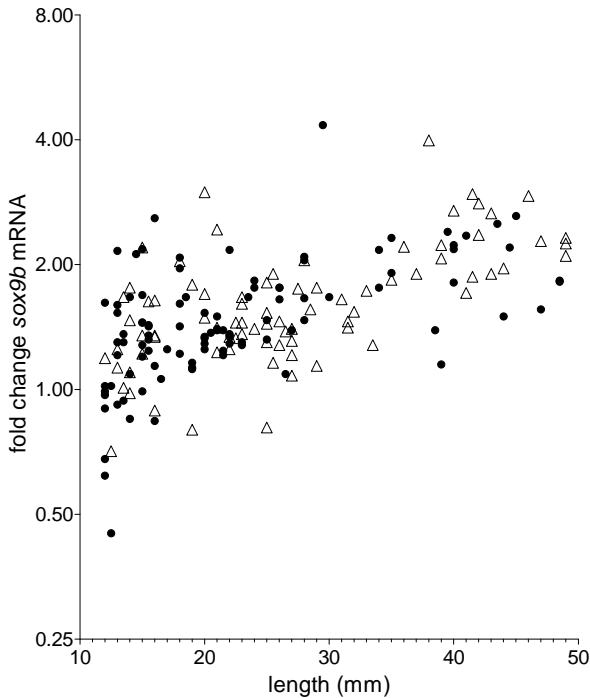

Supplement: Additional file 1 — Figure S1. Gene expression profiles of sox9b in early life stages of mixed sex (open triangles) and all-female (closed circles) Atlantic cod. Data are presented on a logarithmic scale as fold change in mRNA compared to the smallest and youngest fish. [file 1477-7827-10-47-S1.pdf]

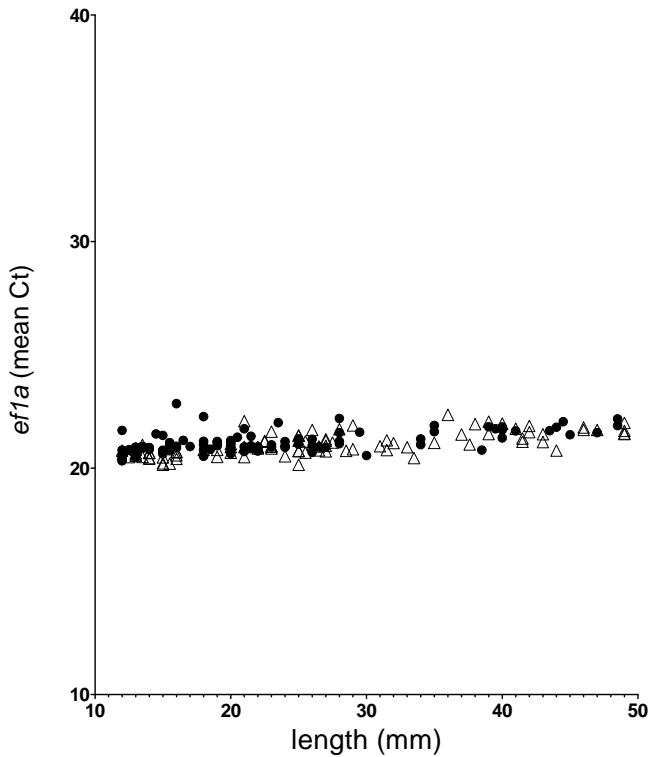

Supplement: Additional file 2 — Figure S2. Scatter plot of Ct values for ef1α mRNA from the mixed sex (open triangles) and all-female (closed circles) Atlantic cod individuals. Data are from quantitative real time PCR. All samples had similar template RNA concentrations loaded into the qPCR reaction. [file 1477-7827-10-47-S2.pdf]

**NJ**

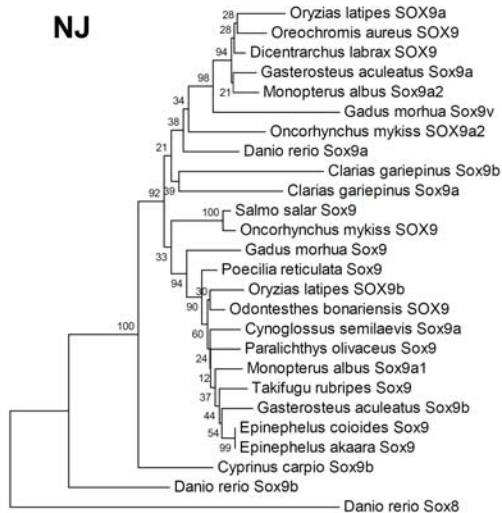

**ML**

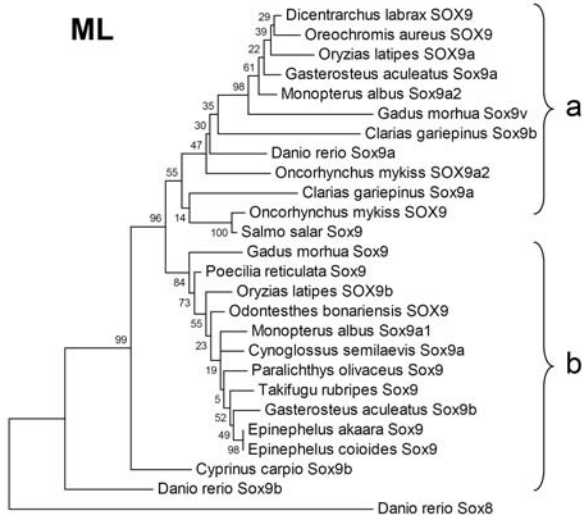

Supplement: Additional file 3 — Figure S3. Phylogenetic analysis of Sox9 amino acid sequences from different teleost species depicted as trees generated by the Neighbor-Joining (NJ) method (left) and Maximum Likelihood (ML) method (right). The bar represents 5% divergence between sequences. The sequences group into two large classes as noted as a and b to the right. The sequences are named according to appearance in the NCBI databank. Only apparently full length sequences were included in the alignment. Clarias gariepinus Sox9a ADJ96868, Clarias gariepinus Sox9b ADJ96869, Cynoglossus semilaevis Sox9a ACY05958, Cyprinus carpio Sox9b AAX56088, Danio rerio Sox9a AF277096, Danio rerio Sox9b AF277096, Danio rerio Sox9b AF277097, Danio rerio Sox8 AAX73357, Dicentrarchus labrax SOX9 CBN81190, Epinephelus akaara Sox9 AAT77677, Epinephelus coioides Sox9 ACT10337, Gasterosteus aculeatus Sox9a AAQ62978, Gasterosteus aculeatus Sox9b AAQ62979, Monopterus albus Sox9a1 AF378150, Monopterus albus Sox9a2 AF378151, Odontesthes bonariensis SOX9 AAP84605, Oncorhynchus mykiss SOX9 BAA24365, Oncorhynchus mykiss SOX9a2 AAG43497, Oreochromis aureus SOX9 ABY66377, Oryzias latipes SOX9b AAX62151, Oryzias latipes SOX9a AAX62152, Paralichthys olivaceus Sox9 ACO40490, Poecilia reticulata Sox9 ABG77973, Salmo salar Sox9 ACN10975, Takifugu rubripes Sox9 AAL32172. The Atlantic cod sequences were obtained through Blast search at The Cod Genome Project web site [57] and translated from the Gadus morhua Sox9 ENSGAUG00000009261 (apparent complete open reading length that include the primer sites used in this communication) and Gadus morhua Sox9v ENSGAUG00000015623. [file 1477-7827-10-47-S3.pdf]
